# An Alternative Diagnostic Method for *C. neoformans*: Preliminary Results of Deep-Learning Based Detection Model

**DOI:** 10.3390/diagnostics13010081

**Published:** 2022-12-28

**Authors:** Ayse Seyer Cagatan, Mubarak Taiwo Mustapha, Cemile Bagkur, Tamer Sanlidag, Dilber Uzun Ozsahin

**Affiliations:** 1 Department of Medical and Clinical Microbiology, Faculty of Medicine, Cyprus International University, TRNC Mersin 10, Nicosia 99010, Turkey; 2 Operational Research Center in Healthcare, Near East University, TRNC Mersin 10, Nicosia 99138, Turkey; 3 DESAM Research Institute, Near East University, TRNC Mersin 10, Nicosia 99138, Turkey; 4 Medical Diagnostic Imaging Department, College of Health Science, University of Sharjah, Sharjah 27272, United Arab Emirates

**Keywords:** artificial intelligence, *C. neoformans*, cryptococcosis, deep learning, diagnosis

## Abstract

*Cryptococcus neoformans* is an opportunistic fungal pathogen with significant medical importance, especially in immunosuppressed patients. It is the causative agent of cryptococcosis. An estimated 220,000 annual cases of cryptococcal meningitis (CM) occur among people with HIV/AIDS globally, resulting in nearly 181,000 deaths. The gold standards for the diagnosis are either direct microscopic identification or fungal cultures. However, these diagnostic methods need special types of equipment and clinical expertise, and relatively low sensitivities have also been reported. This study aims to produce and implement a deep-learning approach to detect *C. neoformans* in patient samples. Therefore, we adopted the state-of-the-art VGG16 model, which determines the output information from a single image. Images that contain *C. neoformans* are designated positive, while others are designated negative throughout this section. Model training, validation, testing, and evaluation were conducted using frameworks and libraries. The state-of-the-art VGG16 model produced an accuracy and loss of 86.88% and 0.36203, respectively. Results prove that the deep learning framework VGG16 can be helpful as an alternative diagnostic method for the rapid and accurate identification of the *C. neoformans*, leading to early diagnosis and subsequent treatment. Further studies should include more and higher quality images to eliminate the limitations of the adopted deep learning model.

## 1. Introduction

Cryptococcus genus involves 70 species; among those, *C. neoformans* and *C. gattii* are classified in the *C. neoformans* species complex, and they are the causative agents of the cryptococcosis, a life-threatening, systemic fungal infection in humans and a wide range of animals [1,2,3].

*C. neoformans* species complex are usually associated with two major infections, pulmonary cryptococcosis and cryptococcal meningitis. Studies mentioned that nearly 67% of pulmonary cryptococcosis, even among immunocompetent persons, can show neurotropism, resulting in cryptococcal meningitis in the central nervous system (CNS) [3].

In addition to lungs and CNS, skin, muscles, joints, bones, liver, kidney, and other organs can be infected too. Non-*C. neoformans* species are *C. laurentii*, *C. uniguttulatus*, and *C. albidus*, and they are rarely associated with infections [3]. *C. neoformans* infections have a global distribution, especially in immunocompromised patients, while *C. gattii* is mainly found in specific geographic regions, such as tropicals and subtropicals, and usually affects immunocompetent individuals [4].

The case of cryptococcosis was first described in the 1890s, before the HIV/AIDS pandemic and effective treatment regimens, and at that time, cryptococcal infections were sporadic. However, cryptococcosis became a severe public health issue in time, which can be observed in both immunosuppressed patients and immunocompetent individuals [4,5]. According to the Centers for Disease Control and Prevention (CDC), CM’s predicted annual case number is around 220,000 worldwide [6]. In 2014, the expected CM case numbers and AIDS-associated deaths were approximately 223,100 and 181,100, respectively, while in 2020, predicted AIDS-associated deaths among CM patients were 580,000 [7]. Although mortality rates with current combined antifungal and antiretroviral treatments still range between 10 and 70%, it is fortunate that human-to-human transmission is not possible [4].

*Cryptococcus* species are abundant in nature, especially in pigeon droppings, soil, and trees [3,4]. The reservoir of *C. neoformans* is mostly pigeon droppings, while *C. gattii* is found chiefly on trees, especially the *Eucalyptus* species [2]. These environments contain salt, creatinine, and high amounts of nitrogen, which support the survival of the yeast. Although pigeons and some bird species are carriers of the yeast, they do not get infected, since their body temperature (41–42 °C) is higher than the optimum growth temperature (37 °C) of the yeast [2].

The main transmission route is the inhalation of small-sized yeast cells. *Cryptococcus* species are found in nature in small-sized and non-capsulated forms. These environmental forms quickly pass through the airways and reach the alveoli [1,5]. In immunocompetent individuals, the yeast cells can be eliminated via innate and adaptive immune system mechanisms, while in most immunocompromised patients, particularly in advanced HIV-positive patients, the cryptococcal cells can escape from the immune system mechanisms. The colonization of the yeast in the lower respiratory tract can result in pulmonary cryptococcosis, an opportunistic and invasive fungal infection, especially in immunocompromised patients. Unfortunately, pulmonary cryptococcosis can be misdiagnosed due to the lack of diagnostic implementations [3].

These aerobic fungi can adapt to human body conditions and easily escape from the immune system via virulence factors [3,5]. The polysaccharide capsule and fungal cell wall’s melanin content are two major virulence factors. The capsule manages to escape from the host’s immune system and inhibits phagocytosis, while melanin protects the yeast from the effects of radiation and reactive oxygen radicals [4,5]. In addition to the ability to transport virulence factors over long distances, ploidy, and polymorphism, the ability to survive in hypoxic environments despite being aerobic, resistance to antifungals, and neurotropism are other hallmarks of pathogenesis [4].

Immunocompetent individuals are mostly asymptomatic. In immunocompromised patients, the symptoms of cryptococcosis can range from mild nonspecific symptoms, such as dyspnea, chest pain, cough, and fever, to life-threatening respiratory failure. Delays in diagnosis and treatment can lead to dissemination to CNS, which is a lethal infection [3]. Ploidy plays a major role in the dissemination of the *C. neoformans* complex from the lungs to the CNS. In pulmonary cryptococcosis, the yeast cells are enlarged and form titan cells, which can survive phagocytosis and are resistant to oxidative stress, and, in this way, can easily disseminate to CNS [3].

The laboratory diagnosis of cryptococcosis is usually based on direct microscopy, culture, histopathologic examination, serology, and molecular techniques. Sputum, bronchoalveolar lavage (BAL), lung biopsy materials, or pleural fluids can be collected as patient samples of pulmonary cryptococcosis patients, while the detection of encapsulated *C. neoformans* in India-ink stained smears of cerebrospinal fluid (CSF) and cultivation of CSF samples in Sabouraud dextrose agar (SDA) are the appropriate diagnostic methods for CM [8]. Additionally, brain heart infusion agar, birdseed extract (BSE) agar (as a selective and differential media), and honey agar media can be used for cultures. Incubations of 28 °C and 37 °C are required for confidential culture results [1].

Despite these techniques being established as the gold standard for diagnosis, recent studies have reported some disadvantages [8]. The culture technique is time-consuming, since it needs approximately seven days of incubation and can reflect colonization rather than infection. Therefore, culture results alone will not be enough for diagnosis and should be confirmed by clinical findings and radiological results for pulmonary cryptococcosis [3]. Cryptococcal antigenemia is an early sign of cryptococcosis and can be a presumption of disseminated cryptococcosis [7]. The United States Food and Drug Administration (FDA) approved a Cryptococcal antigen (CrAg) lateral flow test in 2011, which is a fast, cost-efficient, and easy-to-use test, and it is recommended for cryptococcosis screening [8], especially for HIV/AIDS patients with a CD4 cell count less than 100/µL [9]. However, it should be noted that non-living but capsule compact yeast cells can be detected by CrAg tests too.

The disadvantages of traditional diagnostic methods lead microbiologists to search for new diagnostic methods in the field of microbiology that are faster, cheaper, and more accurate and that guide treatment. Artificial intelligence (AI) implementation in healthcare provides a viable alternative as it has been used in several studies for disease detection, diagnosis, and prediction [10,11,12,13,14]. Implications in AI evolved microbiology to a new diagnostics era, providing many advantages in detecting and identifying microorganisms and leading to optimal treatment strategies [15,16].

This study aims to develop a deep learning approach to detect *C. neoformans* in patient samples as an alternative diagnostic method for a timely and reliable diagnosis.

## 2. Materials and Method

### 2.1. Data and Data Pre-Processing

**Figure 1:**
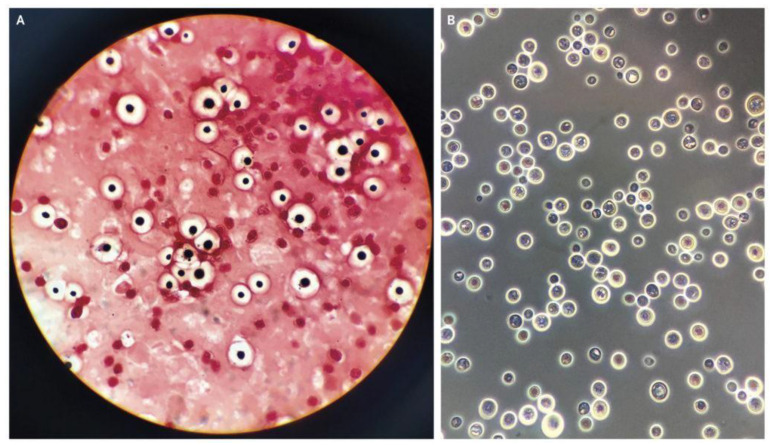
(**A**) Gram staining, (**B**) India ink-stained smear of encapsulated, round yeast cells and *C. neoformans* [17].

**Figure 2:**
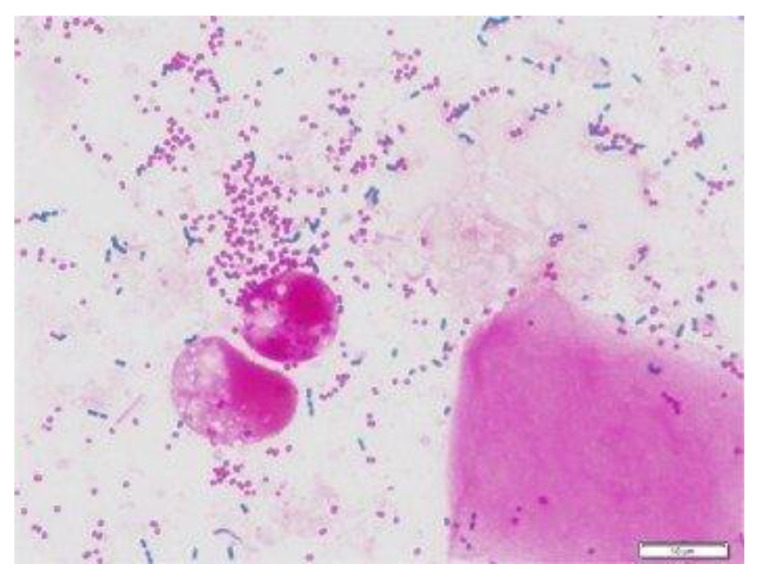
Gram staining of sputum, bacteria cells [18].

The data for this study were sourced by web scrapping the internet for microscopic images of *C. neoformans* and non-*C. neoformans* using keywords, including “India-ink-stained smear of CSF”, “*C. neoformans*”, “stained smear of yeast”, “gram stain *C. neoformans*”, and “India-ink stained *C. neoformans*“. Next, the images were checked for watermarks, copyright inscription, source, and quality and resolution. The images obtained were then subjected to further screening by two teams of expert microbiologists with a combined 40 years of experience in fungal identification. We carried out a double-blinded screening, where the two experts were not informed about each other’s results. Later, the result was compiled and sorted. Images with conflicting results were removed and subjected to a third expert with similar experience and were either added or removed depending on the outcome of the third expert. Finally, the images were grouped into positive and negative. A total of 63 high-quality microscopic images of India-ink stained smears from the CSF samples of the patients were used as the positive dataset (Figure 1). For comparison, microscopic images prepared from CSF, urine, and sputum samples, of which *C. neoformans* was not detected, were used as the negative dataset, as shown in Figure 2.

Data pre-processing is an important step in any deep-learning task. It entails transforming raw data into forms acceptable to the deep learning model. Additionally, because raw data come with arrays of useless components, parts, and features, it is necessary to remove them to promote optimal model performance. The *C. neoformans* microscopic image dataset is limited. Since direct microscopy is not the only diagnostic method and needs clinical expertise for detection, other diagnostic methods are mostly preferred. Therefore, we implemented augmentation techniques to increase the amount of data for the deep learning model, as shown in Table 1. Furthermore, the images were resized to match the size of the image input layer. This helps enhance desired features and reduce artifacts that can bias the deep-learning framework.

In this study, we applied a deep learning approach based on convolutional neural networks (CNN) to identify and classify the microscopic images of *C. neoformans*. In the first instance, literature is reviewed for the microscopic images of India ink-stained smears of CSF, including *C. neoformans*.

Several variants of the dataset were generated using positional and color augmentation techniques while preserving the integrity of the original data. These techniques include shear, rotation, zoom, brightness, shift, and flip. Our history of successfully enhancing imaging data informed the decision to choose a specific range of choices. The shear technique distorts the image to assist in the creation or correction of perception angles and provides a sort of image stretching. In contrast to shear, rotation does not distort an image’s proportions. Rather, rotation changes the angles of the data that appear in the dataset during training. Zoom helps to add new pixels to the image, thereby creating several others. The zero-phase component analysis (ZCA) whitening is a transforming method that decorrelates the image pixel. When utilizing CNN, the spatial arrangement of the pixels must be preserved, and the ZCA does just that. Shifting an image is a geometric transformation that repositions all of its constituent parts relative to one another. The model can be given more variation by shifting the images around to modify the position of the things in the image. This often results in a more generalized model. Flipping allows for the flipping of images in the left–right and up–down directions.

**Figure 3:**
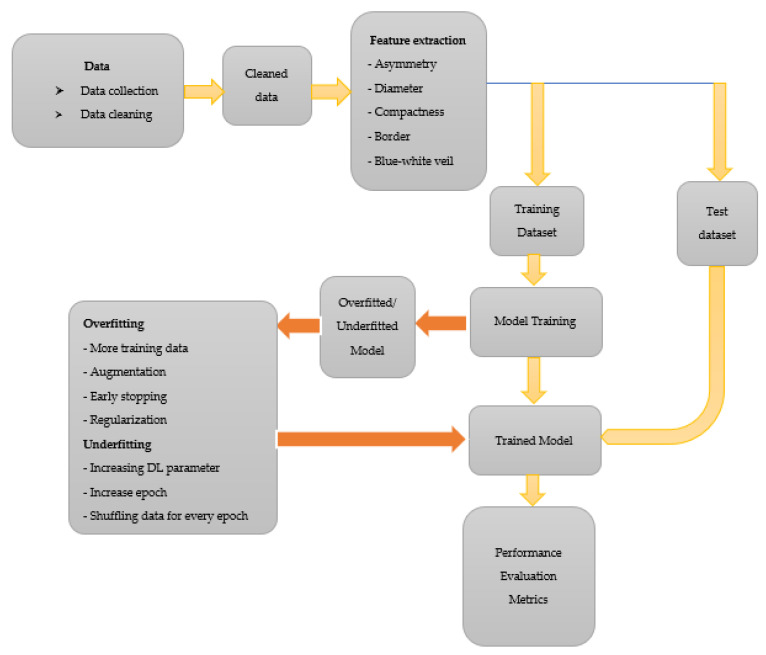
The general procedure followed in classifying images of *C. neoformans*.

The choice of augmenting the data to 1000 is solely due to the combined effort from all authors to avoid over-replicating the data via augmentation. We want to ensure that the ratio of augmentation is within a certain limited range as an increase in the ratio of original and augmented data will abuse the use of augmentation. Let us say we choose to augment the data to 10,000. That is a 0:159 ratio compared with 0:16, the ratio in this study. Over-augmenting data will lead to the model having a similar feature to learn from in data after several epochs, resulting in overfitting. After the augmentation process, the model is trained using 1000 images of *C. neoformans* and 1000 images of species other than *C. neoformans.* The general procedure followed in classifying images of *C. neoformans* is illustrated in Figure 3.

### 2.2. Convolutional Neural Network

One of the most widely used deep learning techniques is convolutional neural networks (CNN), which can take an image as input, assign importance to various objects in the image, and then distinguish between them [19]. Compared to other classification algorithms, a CNN requires significantly less pre-processing [20]. Although filters in primitive methods are often hand-engineered, CNN can learn these filters/characteristics with adequate training [21]. Inspired by the structure of the human brain’s visual cortex, CNNs are designed to mimic how neurons communicate. A CNN may effectively capture an image’s spatial and temporal dependencies by employing the proper filters. Due to the reduced number of parameters and the possibility of reusing weights, the architecture achieves a better fitting to the image dataset [19]. CNN’s job is to simplify the images without losing information vital to making an accurate prediction [22].

#### 2.2.1. Convolution Layer

**Figure 4:**
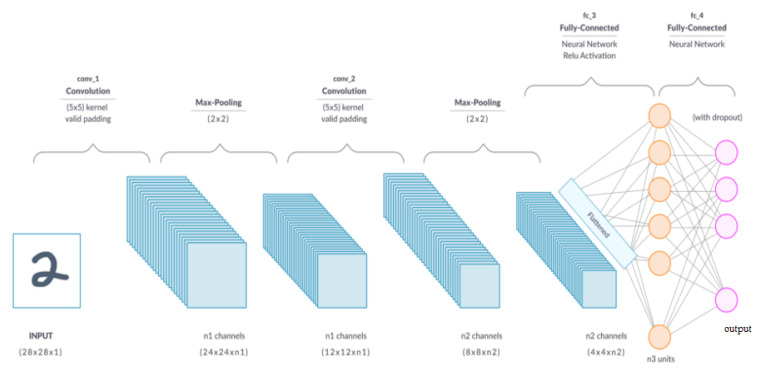
A convolutional neural network [28].

A convolutional layer is the primary building block of a CNN [20]. As can be seen in Figure 4, it comprises a set of filters (or kernels) whose settings will be refined as the training progresses. Extracting high-level characteristics, such as edges from the input image, is the goal of the convolution operation. Low-level features, such as edges, colors, and gradient orientation, are often captured in the first convolution layer [20]. As more layers are added, the architecture can also accommodate high-level characteristics, resulting in a network with a comprehensive grasp of the images in the training set. One conclusion of the operation is that the dimensionality of the convolved feature is lowered relative to the input. In contrast, another outcome is that the dimensionality is increased relative to the input or does not change [23].

#### 2.2.2. Pooling Layer

The pooling layer functions similarly to the convolutional layer in that it reduces the spatial dimensions of the convolved feature [24]. Dimensionality reduction reduces the amount of computer power needed to process the data. In addition, it helps in effectively training the model by extracting dominating features that are rotational and position invariant. Max pooling and average pooling are the two forms of pooling. Max pooling returns the maximum value from the image section covered by the kernel. On the other hand, average pooling returns the mean of all values from the image region covered by the kernel [24]. Max pooling is a noise suppressor as well. It eliminates all noisy activations and conducts de-noising and dimension reduction concurrently. On the other hand, average pooling merely conducts dimension reduction as a noise suppression strategy. Max pooling, therefore, performs significantly better than average pooling. The convolutional layer and pooling layer make up the i-th layer of a CNN. Depending on the complexity of the images, the number of such layers may be expanded to capture even more low-level details at the expense of higher computing power [25].

#### 2.2.3. Fully Connected Layer

The final layer of a convolutional neural network is the fully connected layer, often known as the hidden layer. This layer consists of both affine and non-linear functions [26]. The fully connected layer of the neural network is essentially a feed-forward network. The output of the final pooling or convolutional layer is sent into the fully linked layer after being flattened. A layer with complete connectivity multiplies the input by a weight matrix and then adds a bias vector [27].

### 2.3. VGG16

**Figure 5:**
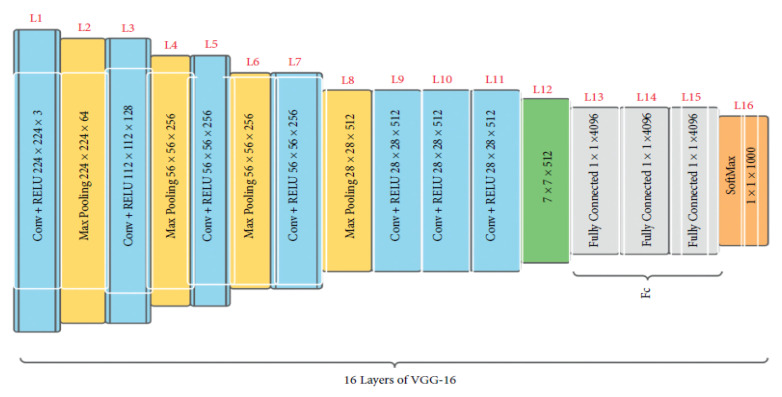
The architecture of VGG16 [32].

Initially presented in 2015 [29], VGG16 is a convolutional neural network trained with data from a subset of ImageNet, a database of over 14 million images split into over 22,000 classes [29]. VGG16 is a popular model for image classification and object detection tasks [30]. Additionally, producing high accuracy in image classification tasks is VGG19, a version of VGG16 [31]. There are 16 weighted layers, hence the “16” in VGG16. VGG16 includes 21 layers—13 convolutional layers, 5 Max Pooling layers, and 3 Dense layers—but only 16 weight layers (the layer in which parameters can be trained). VGG16 accepts 224 × 244 tensors with three RGB (red, green, and blue) channels for input. What makes VGG16 stand out is that instead of using a wide variety of hyper-parameters, the authors standardized on a 3 × 3 filter with stride 1 for convolution layers and a 2 × 2 filter with stride 2 for padding and max pool layer. Figure 5 shows that the convolution and max pool layers are consistently organized across the board. There are 64 filters in the Conv-1 layer, 128 in the Conv-2 layer, 256 in the Conv-3 layer, and 512 in the Conv-4 and Conv-5 layers. After the stack of convolutional layers comes the three fully connected (FC) layers, the first two of which have 4096 channels each and the third of which conducts 1000-way ILSVRC classification and so has 1000 channels (one for each class). The final layer is the soft-max layer [29]. The layers and the total number of parameters of the sequential model are displayed in Table 2. This system is built with 15 layers for more accuracy. Then, convolutional and pooling layers were added. Consequently, the overall number of parameters increased.

### 2.4. Confusion Matrix

**Figure 6:**
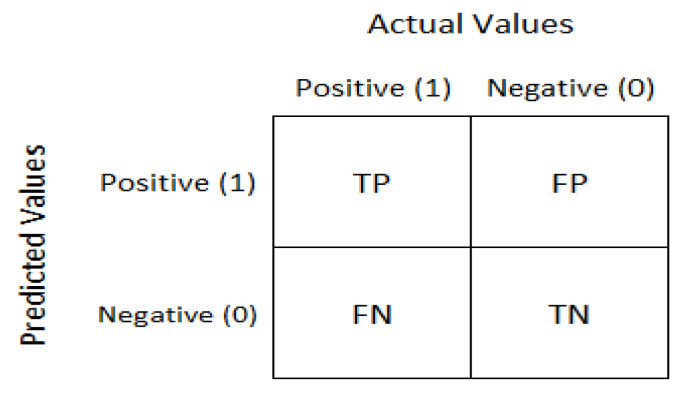
The confusion matrix [33].

The confusion matrix is used for a given set of test data to evaluate how well a model performs. It is used to determine if the true values for test data are known. As depicted in Figure 6, the confusion matrix evaluates the performance of the models when making predictions on test data and reflects the efficiency of our classification model. It indicates not only the error made by the model but also other errors, including type-I or type-II. The following terminologies depict the component of a confusion matrix [10].

- True negative (TN): model has given prediction negative, and the real or actual value was also negative.
- True positive (TP): the model has predicted positive, and the actual value was also positive.
- False negative (FN): the model has predicted negative, but the actual value was positive; it is also called a Type-II error.
- False positive (FP): the model has predicted positive, but the actual value was negative. It is also called a Type-I error.

## 3. Result and Discussion

This study uses a deep learning framework to detect *C. neoformans* using microscopic images of India-ink-stained smears of CSF. Images that contain *C. neoformans* are designated positive, while others are designated negative throughout this section. Before model training, the dataset was split into a 70% training set and a 30% test set. Of the 70% training set, a further 20% was used as a validation dataset. Model training, validation, testing, and evaluation were conducted using frameworks and libraries, including Jupyter notebook, TensorFlow, Keras, Pandas, NumPy, Matplotlib, and Seaborn. The implementation tools and framework used in this study were developed on a personal computer (PC) with Windows 10 Pro, 11th Gen Intel (R) Core (TM) i7-11700KF @ 3.60 GHz (Gigahertz) 3.60 GHz processor, 64.0 GB (Gigabyte) installed RAM (Random Access Memory), 64-bit operating system, and NVIDIA GeForce RTX (Ray Tracing Texel eXtreme) 3070 GPU (graphic processing unit) card to meet the training of deep neural network workload requirements. Python was the programming language of choice throughout the study.

The study proves that the deep learning framework can help detect *C. neoformans* in the microscopic image of India-ink-stained smears of CSF. The state-of-the-art VGG16 model produced an accuracy and loss of 86.88% and 0.36203, as shown in Table 3. The accuracy and loss values show how the VGG16 model performed in detecting and classifying the images into positive and negative. The accuracy indicates how effective the model is at generalizing unseen data, while the loss indicates fewer errors were made. Loss is the penalty for a wrong decision. It depicts the distance between the true value and the predicted value. The greater the loss, the more enormous the error made by the model on the data. The values of loss range from 0–1, where 0 indicates that the model’s prediction is perfect, while a loss of 1 means the model is making a terrible prediction.

**Figure 7:**
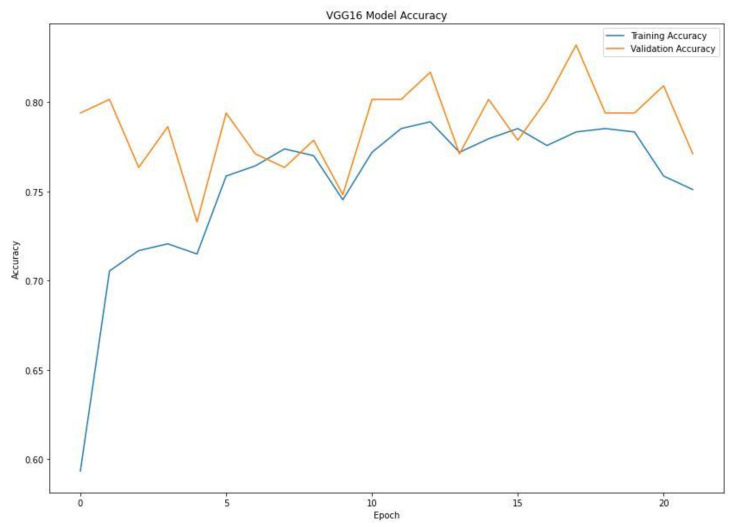
Training and validation accuracy of the VGG16 model.

**Figure 8:**
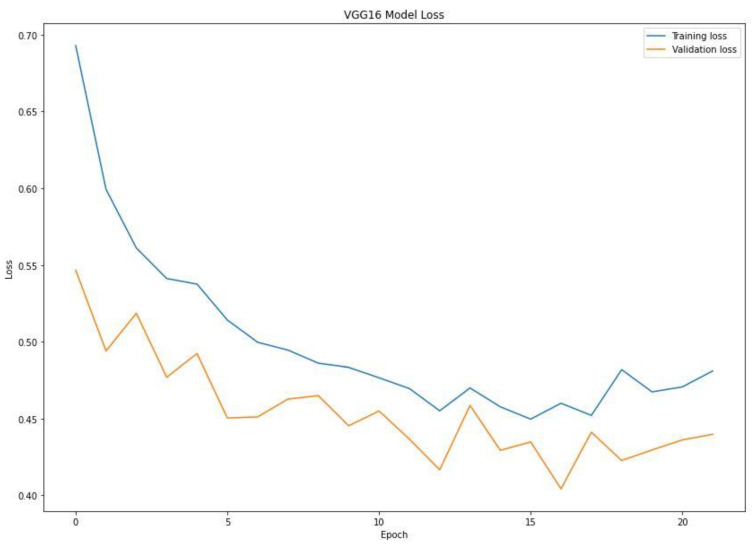
Training and validation loss of the VGG16 model.

When training a machine learning model, one of the main things to avoid is overfitting. This is when the model fits the training data so well that it cannot generalize and make accurate predictions for data it has not seen before (test data). Metrics on the training data indicate how well the model is progressing in terms of its training, but it is the metrics on the validation data that provide the measure of the quality of a model—how well it can make new predictions based on data it has not seen before. Figure 7 shows the accuracy obtained during training and validation. The training and validation accuracy in a typical learning curve is expected to increase with each epoch. The VGG16 model accuracy increased with a corresponding validation accuracy. This generates a good fit void of overfitting and underfitting. The training and validation loss are the terms used to measure how a deep learning model fits the training and validation data. This indicates the performance of the model on the data. As indicated in Figure 8, the training and validation loss indicates how the VGG16 model fits the training and validation data and identifies which aspect needs tuning. The VGG16 generated a relatively stable good fit as the training and validation loss decreased and gradually stabilized. A high loss value usually means the model produces erroneous output, while a low loss value indicates fewer errors in the model. In addition, the loss is generally calculated using a cost function, which measures the error in different ways. Because of the nature of the study (binary classification), we adopted binary cross-entropy.

Aside from accuracy and loss, the performance of a deep learning model can be measured using other metrics. These metrics provide a much more robust evaluation of the model. Accuracy alone is not enough to measure a model’s performance [10]. Precision is a machine learning metric that indicates the quality of a positive prediction made by the model. It provides insight into the number of true positives predicted by the model. Table 4 shows the mean performance metrics of the VGG16 model. With a precision of 90.00% and 85.00% for positive and negative images, the VGG16 shows its capability to effectively identify and classify smear images containing *C. neoformans* and those without it. The sensitivity of 83.00% and 91.00% for both positive and negative images indicate the model’s ability to correctly predict the proportion of true positives that are correctly predicted.

**Figure 9:**
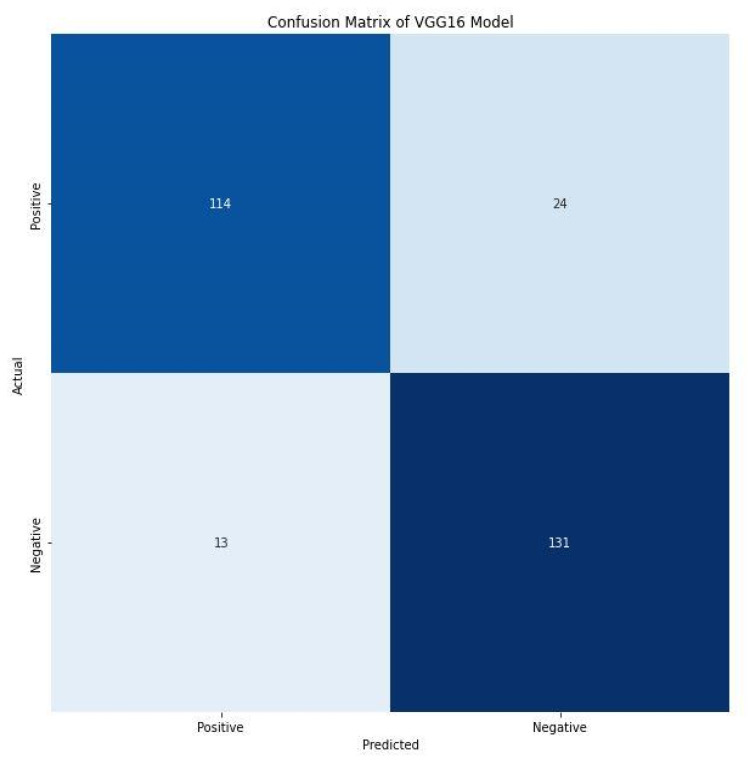
Confusion matrix of VGG16 model.

Furthermore, the F1 score of 86.00% and 88.00% for positive and negative images indicate the harmonic mean of precision and recall. It combines precision and recalls into a single number using the following formula. A confusion matrix prints the correct and incorrect values in the number count. It provides an understanding of data visualization and gives insight not only into the errors made by a classifier but, more importantly, the types of errors being made. Figure 9 shows the confusion matrix of the VGG16 model. The VGG16 model correctly detects and classifies 245 images, 114 of which were positives, while 131 were negatives. This indicates good precision and the applicability of deep learning frameworks for the detection of *C. neoformans* in smear images. However, 37 images were misclassified, of which 24 were falsely classified as positives and 13 as false negatives. The performance evaluation metrics generated substantive outcomes that can aid the rapid detection of *C. neoformans* and aid in managing immunocompromised patients.

We compared the performance of our model with two state-of-the-art pre-trained models—ResNet50 and InceptionV3. ResNet-50 is a convolutional neural network that is 50 layers deep. It is a smaller version of ResNet 152 and uses a deeper network to avoid poor accuracy. Each convolution block has three convolution layers, and each identity block has three convolution layers. The ResNet50 has over 23 million trainable parameters [34]. InceptionV3, also called GoogleNet, is CNN architecture from the Inception family that makes several improvements, including label smoothing, factorized 7 × 7 convolutions, and an auxiliary classifier to propagate label information to lower the network. The InceptionV3 is a superior version of the InceptionV1. It has 42 layers and a lower error rate than its predecessors [35].

**Figure 10:**
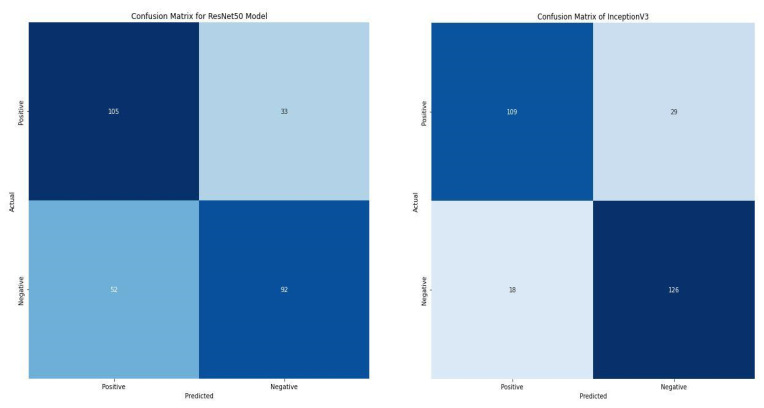
Confusion matrix of ResNet50 and InceptionV3.

Compared with the two state-of-the-art pre-trained models, the VGG16 model significantly outperformed them with accuracy and loss of 86.88% and 0.36203, as shown in Table 5. Additionally, the model correctly classifies 245 images as positive and negative. In contrast, the ResNet50 and InceptionV3 correctly classify 197 and 235 images. Furthermore, ResNet50 and InceptionV3 misclassify 85 and 47, respectively. In retrospect, the VGG16 only misclassifies 37 images, as shown in Figure 10.

Conventional diagnostic methods for the identification of *Cryptococcus* species have been used widely in the past. However, some disadvantages of these techniques caused delayed treatment and increased mortality. Several studies reported the disadvantages of cryptococcal cultures; living cryptococcal cells in CSF samples are required [8], samples taken from patients under systemic antifungal treatment require a longer incubation period [3], and positive culture results should be confirmed by patients’ clinical findings [3]. Although serologic tests for the detection of cryptococcal antigens are meaningful in laboratories with inadequate medical equipment, cryptococcal antigen detection of blood serum sensitivity and specificity are reported as 83–100% and 72–100%, respectively. In CSF samples, the sensitivity of serologic tests was within 80–100%, while specificity was found to be within 82–100% [8]. The detection of *C. neoformans* from respiratory system samples of diagnosed pulmonary cryptococcosis cases by multiplex RT PCR showed 90.7% sensitivity and 100% specificity [3]. Huston and Mody reported that the low fungal load and prozone effect could lead to false negative results in latex agglutination tests, where rheumatoid diseases; the presence of some other microorganisms, such as *Trichosporon beigelii*; the effect of some chemicals, such as disinfectants; and an extended waiting period of serum samples can lead to false positive results [36].

In recent years, opportunistic fungal infection incidence has increased dramatically due to the frequent use of broad-spectrum antibiotics and immunosuppressive medications [37]. Cryptococcus spp. is an opportunistic fungus, susceptible to polyenes, flucytosine, and azoles [38]. Polyenes decrease the ergosterol content of the plasma membrane, while azoles inhibit ergosterol biosynthesis, and flucytosine blocks DNA synthesis [39]. In some conditions, lifelong therapy is required [35].

Usually, combined Amphotericin B, flucytosine, and fluconazole therapy are applied against CM, which is effective in immunocompetent populations [37]. However, the excessive usage of antifungals in agriculture and medicine induced the emergence of antifungal-resistant strains of Cryptococcus spp., which is one of the major difficulties in CM treatment [38].

Besides the resistance challenges, these drugs are expensive, and various side effects, such as toxicity, are reported [37]. Therefore, cryptococcosis is still a public health concern, and new antifungal drug developments or other therapeutic strategies are required [39].

The International Treatment Preparedness Coalition (ITPC) published a global strategic plan, ‘Ending Cryptococcal Meningitis Deaths by 2030’, which aims to reduce CM-related deaths by 90% by 2030 from the 2020 baseline [40]. This goal can only be achieved if diagnosis, treatment, and preventive screening programs are implemented immediately [7]. Machine learning applications in microbiology are promising for accurate and timely diagnosis, and the state-of-the-art VGG16 model, which is applied for the first time in this study, showed similar accuracy (86.88%) to some of the diagnostic methods that have been in practice for many years.

Species–specific fungal diagnosis needs further diagnostic tests after the detection of yeast-like fungi under the microscope. Usually, cultivation, biochemical tests, molecular tests, and sequencing are employed for species–specific identification. However, these techniques prolong the time period required for the results and are expensive. On the other hand, our CNN method, which can detect *C. neoformans* based on basic microbiological staining (India ink) of the patients’ samples, enables the diagnosis only in a few minutes [41].

## 4. Conclusions

Cryptococcosis was considered an uncommon disease before the frequent use of immunosuppressive therapy and the emergence of the HIV/AIDS pandemic. The dramatic increase in the incidence of Cryptococcus spp. also increased the interest of researchers to understand the morphology, pathogenesis, diagnosis, and treatment strategies of this fungi [10].

Research in machine learning is evolving rapidly, and applications in microbiology evolved this field to a new era [10]. This study is a pioneer in the literature, since it is the only study that is designed to directly detect *C. neoformans* in India-ink-stained smears of CSF samples collected from patients. The preliminary results of this study demonstrate that deep learning frameworks can provide an effective and accurate choice for *C. neoformans* detection, thereby leading to early diagnosis and subsequent treatment. The study’s outcome also demonstrates that with minimal training and a small test dataset, an accuracy of 86.88% was achieved by the VGG16 model. At the same time, other metrics, including precision, sensitivity, and F1 score evaluated, show the reliability of the result obtained.

Deep learning methods, especially CNN, have shown human-level performance in the case of large amounts of training data; however, since the microscopic examination is not the only diagnostic method for the diagnosis of *C. neoformans*, the microscopic image datasets are limited. The lack of fungal image libraries also makes the data collection process difficult. Therefore, the photographic documentation of the *C. neoformans* images can be useful to obtain high quality images and many of them, and we can create a microscopic image dataset.

Further studies should include more and higher quality images to eliminate the limitations of the adopted deep learning model.

## Figures and Tables

**Table 1:** Distribution of dataset.

		Original Number of Dataset	Augmented Number of Dataset
1	Positive	63	1000
2	Negative	63	1000

**Table 2:** VGG16 layers and parameters.

Layers	Outcome Structure	Number of Parameters
Conv1	(224 × 224 × 64)	1792
Conv2	(224 × 224 × 64)	36,928
MaxPool	(112 × 112 × 64)	-
Conv1	(112 × 112 × 128)	73,856
Conv2	(112 × 112 × 128)	147,584
MaxPool	(56 × 56 × 128)	-
Conv1	(56 × 56 × 256)	295,168
Conv2	(56 × 56 × 256)	590,080
Conv3	(56 × 56 × 256)	590,080
MaxPool	(28 × 28 × 256)	-
Conv1	(28 × 28 × 512)	1,180,160
Conv2	(28 × 28 × 512)	2,359,808
Conv3	(28 × 28 × 512)	2,359,808
MaxPool	(14 × 14 × 512)	-
Conv1	(14 × 14 × 512)	2,359,808
Conv2	(14 × 14 × 512)	2,359,808
Conv3	(14 × 14 × 512)	2,359,808
MaxPool	(7 × 7 × 512)	-
Flatten-Layer	(25,088)	-
Fully connected layer Dense-1	(4096)	102,764,544
Fully connected layer Dense-2	(4096)	16,781,312
Dropout	(4096)	-
Softmax	(1)	4097

Total number of parameters: 134,264,641. Parameters: 134,264,641; Non-trainable Params: null.

**Table 3:** Summary of VGG16 model.

Large Number	Parameter	Test Accuracy	Test Loss
16	134,264,641	86.88%	0.36203

**Table 4:** Mean performance metrics.

	Precision	Sensitivity	F1 Score	TP	TN	FP	FN
Positive	90.00%	83.00%	86.00%	114	131	24	13
Negative	85.00%	91.00%	88.00%				

**Table 5:** Performance evaluation of the state-of-the-art pre-trained model.

		Prec. %	Sens. %	F1 Score %	TP	TN	FP	FN	Acc. %	Loss
VGG16	Pos.	90.00	83.00	86.00	114	131	24	13	86.88	0.36203
	Neg.	85.00	91.00	88.00						
ResNet50	Pos.	86.00	79.00	82.00	105	92	33	52	83.33	0.39312
	Neg.	81.00	88.00	84.00						
InceptionV3	Pos.	67.00	76.00	71.00	109	126	29	18	69.86	0.55700
	Neg.	74.00	64.00	68.00						

Positive = Pos. Negative = Neg. Precision = Prec. Sensitivity = Sens. Accuracy = Acc.

## Data Availability

Data is available upon the requests.
